# In Vitro Evaluation of the Antibacterial Activity of Three Root Canal Sealers

**Published:** 2010-02-20

**Authors:** Yazdan Shantiaee, Omid Dianat, Anoosheh Janani, Golbarg Kolahi Ahari

**Affiliations:** 1. Department of Endodontics, Iranian Center for Endodontic Research, Dental Research Center, Dental School, Shahid Beheshti University of Medical Sciences, Tehran, Iran.; 2. Dental Student, Dental School, Shahid Beheshti University of Medical Sciences, Tehran, Iran.; 3. General Linguistics, Tehran, Iran.

**Keywords:** AH26, Antibacterial activity, Apexit, Endodontic sealers, ZOE sealer

## Abstract

**INTRODUCTION:**

Antibacterial activity is one of the desirable properties of an ideal sealer. This study aimed to compare the antimicrobial effect of three different sealers, i.e. resin (AH26), calcium hydroxide (Apexit) and zinc oxide eugenol (ZOE) based sealers.

**MATERIALS AND METHODS:**

Direct contact test with agar diffusion was used in this in vitro study. The freshly mixed sealers were AH26, Apexit and pure ZOE. They were prepared according to manufacturer’s instruction and placed in prepared wells of 30 agar plates inoculated with Streptococcus (S) mutans and Prevotella (P) melaninogenicus (15 samples for each microorganism). All plates were incubated for 7 days (196 hours) at 37˚C under anaerobic conditions, and zones of inhibition were measured after 3 days, 5 days and 7 days. The data were analyzed using Kruskal-Wallis and Friedman tests.

**RESULTS:**

In all determined intervals, the antibacterial activity of AH26 was significantly greater than other test materials (P<0.001). ZOE sealer had moderate effect on test microorganisms, whilst Apexit showed the lowest antibacterial activity on S. Mutans and no antibacterial activity on P. melaninogenicus.

**CONCLUSION:**

The ascending sequence of bacterial growth inhibition zones was as AH26>Pure ZOE>Apexit.

## INTRODUCTION

One of the important goals of endodontic treatment is comprehensive obturation of root canals. The success of obturation is directly related to adequate removal of microorganisms and their by-products through mechanical root canal instrumentation, antibacterial irrigation, adequate filling of the root canal space, and use of inter-appointment antimicrobial dressings (e.g. calcium hydroxide) when necessary [1][2][3]. However, these procedures do not completely sterilize the root canal system [4]. The proliferation and growth of remaining intra-canal microorganisms may destroy periapical tissues and results in periapical pathosis [5]. Furthermore, if the access cavity is not sufficiently sealed, bacteria may penetrate into an obturated root canal within few days; persisting or re-infecting bacteria may induce or sustain apical periodontitis [6]. Therefore, endodontic filling materials should be antibacterial/antimicrobial[7]. Adding anti-microbial agents to root canal sealers is a method which can add antimicrobial properties to the sealers [1].

Today, numerous root canal sealers are available based on various formulas, such as epoxy resin sealers, calcium-hydroxide-based materials, and zinc oxide eugenol (ZOE) cements with and without paraformaldehyde additions [7]. Improved calcium hydroxide-based sealers have been proposed for permanent seal of the root canal system. Calcium hydroxide compounds are widely used; they have excellent bactericidal action due to their alkalinity [8], they mediate degradation of bacterial lipopolysaccharides, induce healing by hard tissue formation, and control inflammatory root resorption. Resin-based root canal filling materials have steadily gained popularity and are now accepted and used for anterior and posterior teeth. The bonding systems have improved sealing ability, which explains the resistance of some materials to bacterial penetration [9]. ZOE has some antimicrobial activity because the zinc oxide and eugenol components can diffuse through the agar [10]. However, the controversies around antimicrobial effects of sealers on common isolated bacteria in infected teeth, complicates clinicians decision in choosing a suitable sealer [11][12][13][14][15][16]. Since different bacteria may vary in their sensitivity to one material, using more than one type of the bacteria in the evaluation of antibacterial activity of the sealers is required [11]. It has been shown that antimicrobial activity of sealers depends on the time interval between mixing and testing. Most sealers exhibited antibacterial activity when freshly mixed; this decreases over time[12]. Kaplon et al. suggested the importance of evaluating the antimicrobial effect of sealers over longer intervals [11][12].

The purpose of the current study was to compare the antimicrobial effect of three different types of sealers on two types of isolated root canal microorganisms: Streptococcus (S) mutans and Prevotella (P) melaninogenicus at three different time intervals.

## MATERIALS AND METHODS

The studied root canal sealers were AH26 (Dentsply De Trey, Konstanz, Germany), Apexit (Ivoclar Vivadent Inc., NY., USA), and ZOE (Kemdent work Ltd, England). They were prepared according to the manufacturers’ instructions.

The microorganisms used in this study were one facultative anaerobic, S. mutans; and one obligate anaerobic, P. melaninogenicus.

S. mutans was maintained in Tryptic Soy Broth (TSB) (Difco Laboratories, Detroit, Michigan, USA) and P. melaninogenicus in prereduced anaerobically sterilized brain heart infusion broth (BHIB), supplemented with hemin (5 mg/L) and menadione (0.5 mg/L). The turbidity of the inoculum, prepared in TSB or BHIB, was adjusted to the turbidity of 0.4 McFarland Standard. For the agar diffusion test, the following mediums were used: Mitis-salivaris agar plate (MSA; Difco, Detroit, Mich., USA) for S. mutans, and brucella blood agar plate for P. melaninogenicus. Bacteria inoculation was performed using sterile cotton tipped applicator, and four wells with 4 mm depth and 6 mm width (diameter) were punched in each agar plate and filled with the freshly prepared sealers. Agar plates inoculated with both bacteria were placed in an anaerobic cabinet supplied with CO_2_ 5%, H2 10% and N2 85% at 37˚C for 1 week. Positive control plates were streaked with bacteria, but no root canal sealer was used. Subsequently, the diameters of bacterial inhibition zones were measured and recorded for each sealer after 3 (72 hours), 5 (120 hours) and 7 days (168 hours) with the diameter of 6 mm as the cut-off value.

Five agar plates were used for each bacterial strain tested. All assays were repeated three times to ensure reproducibility. Kruskal-Wallis and Friedman tests were used for statistical analysis. Multiple comparisons carried out with mann-whitney and wilcoxon tests after bonferroni adjustment of α.

## RESULTS

The results have been summarized in Table 1 and Table 2. All tested sealers were distinctly different from each other in their antimicrobial activity. AH26 demonstrated large zones of inhibition against two bacteria tested. ZOE had moderate antibacterial activity against S. mutans and P. melaninogenicus. Apexit showed antibacterial activity only on S. mutans. Moreover, Apexit exhibited the least zone of inhibition against S. mutans compared to the others. Positive control plates showed bacterial growth in all cases.

**Table 1:** Mean (SD) diameter of S. mutans zones of growth inhibition with 3 root canal sealers.

**Sealer**	**3^rd^ Day**	**5^th^ Day**	**7^th^ Day**	**P value**
**AH26**	33.13 (4.68)	34.93 (6.96)	34.93 (6.96)	0.052
**Apexit**	10.40 (0.73)	12.066 (1.43)	12.066 (1.43)	0.036
**Pure ZOE**	22.2 (1.01)	23.33 (1.17)	23.33 (1.17)	0.008
**P value**	0.001	0.001	0.001	

**Table 2:** Mean (SD) diameter of P. melaninogenicus’s zones of inhibition with 3 root canal sealers

**Sealer**	**3^rd^ Day**	**5^th^ Day**	**7^th^ Day**	**P value**
**AH26**	38.5 (2.168)	39 (2.85)	39 (2.85)	0.334
**Apexit**	0.0 (0.0)	0.0 (0.0)	0.0 (0.0)	-
**Pure ZOE**	16.8 (2.24)	15.66 (2.43)	15.66 (2.43)	0.016
**P value**	0.001	0.001	0.001	

According to this study, AH26 sealer had the greatest antibacterial action and Apexit, the least (P<0.05).

In summary the sequence of the antibacterial activity of studied sealers was AH26>Pure ZOE>Apexit.

## DISCUSSION

There are an increasing number of reports regarding anaerobes infecting the root canal system, particularly when the infection has been long standing. Anaerobic bacteria especially, may be well adapted to survive in necrotic pulp and dentinal tubules where the blood and oxygen supply is limited or absent. Facultative anaerobic microorganisms may interact with strict anaerobes, causing changes in nutritional relationships and shifts into the redox and oxygen tension, which determine microbial survival relationship [7]. Antibacterial activity of root canal sealers in these microorganisms may assist in controlling infection. In addition, the great prevalence of facultative anaerobes and obligate anaerobes in unsuccessful endodontic treatment [3] necessitates the use of bactericidal root canal sealers.

The agar diffusion method has been widely employed to investigate the antimicrobial activity of dental materials. However, this procedure is influenced by two factors: the materials’ microbial toxicity as well as the materials’ diffusion and affinity in the culture medium [23]. A material that easily diffuses will produce larger zones of inhibition of bacteria [4][17][18][19][20][21][22][34]. In addition, a disadvantage of this method is that it cannot distinguish between bacteriostatic or bacteriocidal properties of the materials. Several limitations such as lack of standardization of inoculums density, adequate culture medium, agar viscosity, plate storage condition, size and number of specimens per plate, and time and temperature of incubation exist [20][22]. Standardization of these factors allows us to reach more conclusive results and exclude the numerous variables existing in-vivo.

In this study, the freshly processed root canal sealers were immediately placed into agar plates.

Because of various temporary or permanent by products, dental materials should be tested immediately after mixing and when final chemical setting stage has been reached. Root canal sealers are used in patients when freshly mixed (incompletely set); thus it is likely that after their clinical application local responses are provoked by leaching components that have partially set or not set at all. However, after setting, toxic ingredients may still be released from the materials. The difference in antimicrobial patterns of various materials may be related to the degree or time taken to set.

Our data suggests substantial differences in antimicrobial effect among root canal sealers. Several authors have studied the antibacterial properties of various root canal sealers against different microorganisms [1][4][11][12][13][14][15][16][17][18][19][20][21][22][23]. However, the results were controversial [12][27]. The sensitivity of antibacterial properties depends on different factors i.e. type of materials, inoculated bacteria, test method, and interval times. The sealers evaluated in this study showed different inhibitory effects depending on the type of root canal sealers and bacterial strains tested. Overall, AH26 proved to be the most effective against the endodontic pathogens. AH26 had the largest inhibition zone in comparison with other ones which is in agreement with Mohammadi and Yazdizadeh, Tabrizizadeh and Mohammadi, and al-Khatib et al. studies [13][14][17]. Conversely, some studies showed that AH26 had the least or no antimicrobial effect [1][27][28][29]. Zhang showed that AH Plus has no significant antibacterial activity after the first hour[27]. Difference in microorganism strains used and the testing methods may be the main reasons of these controversies. Siquera suggested the method of study e.g. agar diffusion may be the main reason for the variance found with our results and other research [21].

Our results showed an increase of inhibition zone of AH26 after third day and optimal antibacterial activity after 5 days and similar values after 7 days. However, other studies have shown that AH26 had its optimal antimicrobial activity after 72 hours and the inhibition zone subsequently reduced [23]. Grossman’s study in 1980 also showed that AH26 had no antimicrobial effect after the fifth day[29]. The findings of current study also revealed that inhibition zone of ZOE in obligate anaerobic cultivating dishes had reduced after the third day, although it had increased in facultative anaerobic ones. It seems that ZOE is more appropriate for elimination of aerobic and facultative anaerobic bacteria compared to obligate anaerobic ones.

The antimicrobial effect of root canal sealers containing ZOE cement has been attributed to free eugenol liberated from the material [27]. Eugenol, a phenolic compound, is effective against mycotic cells in their vegetative form [7].

Resin-based sealers such as AH26 have been shown to be antimicrobial. The antimicrobial effect of resin-based sealers may be related to bisphenol A diglycidyl ether which was previously identified as a mutagenic component of the resin based material[24]. In addition, formaldehyde release in the polymerization process may also assist its antimicrobial properties [19][25].

Calcium hydroxide based sealer was shown to be appropriate for elimination of bacteria. Calcium hydroxide antimicrobial property is due to the ionization process which releases OH־ ions, causing an increase in PH. A PH>9 may reversibly or irreversibly inactivate cell membrane enzymes of microorganism, resulting in a loss of biological activity [1]. Apexit released calcium hydroxide and showed only slight toxicity in the fresh state. However, this sealer is ineffective against obligate anaerobic bacteria, P. melaninogenicus. Zhang et al. also showed poor antibacterial activity for Apexit in comparison to six other sealers against E. Faecalis [26]. The slow and low concentration of OH־ ions and resistance of microorganisms to alkaline environment may explain this observation.

Further studies by other suggested methods such as direct contact test is recommended for antibacterial evaluation of Apexit. Poor antimicrobial activity of Apexit against anaerobic bacteria, frequently found in infected canals, has made it unpopular for root canals treatments. The most appropriate sealer for root canal therapies should be chosen based on various characteristics, including antimicrobial activity. It must be emphasized that root canal sealers with strong antibacterial activity have frequently been found to induce adverse effects during and after treatment; they were also cytotoxic or even mutagenic [19][24][25][27][30]. There is a controversy about antimicrobial effect of sealers after several days[1][10][31][32][33].

Further studies are recommended to achieve more reliable results in this field.

An ideal root canal sealer should have good antimicrobial activity as well as low toxicity for surrounding tissues [31]. Endodontists should accept that biocompatibility (e.g. cytotoxicity, genotoxicity, mutagenicity, carcinogenicity, as well as antimicrobial efficacy) [31] is at least as important as physical and chemical properties of root canal sealers.

## CONCLUSION

Zones of bacterial growth inhibition for both bacterial species tested were observed in ascending order AH26>Pure ZOE>Apexit. Further in vivo and in vitro studies are advised.
